# Efficacy of high-intensity laser therapy for patellofemoral pain: a systematic review and meta-analysis

**DOI:** 10.1186/s12998-026-00653-z

**Published:** 2026-06-12

**Authors:** Hernán Andrés de la Barra Ortiz, Nivaldo Antonio Parizotto, Claudio Hernán Chamorro Lange, Richard Eloin Liebano

**Affiliations:** 1 Faculty of Rehabilitation Sciences, School of Physical Therapy, Exercise and Rehabilitation Sciences Institute, Universidad Andres Bello, Avenida Fernández Concha 700, Las Condes, 7591538 Santiago, Chile; 2 Institute for Advanced and Strategic Studies, Federal University of São Carlos (UFSCar), São Paulo, Brazil; 3 Department of Rehabilitation Sciences, University of Hartford, West Hartford, CT USA

**Keywords:** Lasers, Laser therapy, High-intensity laser therapy, Patellofemoral pain syndrome, Patellofemoral joint, Pain management

## Abstract

**Introduction:**

Patellofemoral pain (PFP) is a common cause of anterior knee pain, particularly among physically active individuals. High-intensity laser therapy (HILT) has shown analgesic effects in several musculoskeletal conditions; however, its role in PFP remains unclear.

**Objective:**

To evaluate the effects of HILT in people with PFP.

**Method:**

Randomized controlled trials (RCTs) evaluating HILT for PFP were identified through searches in PubMed, Scopus, Web of Science, CINAHL, MEDLINE, ScienceDirect, the Cochrane Library, and PEDro, with additional screening of reference lists and gray literature (last search: May 1, 2026). The primary outcome was pain intensity, while functional outcomes were considered secondary. Risk of bias was assessed using the Cochrane RoB 2 tool. Meta-analyses were conducted using standardized mean differences (SMDs) with 95% confidence intervals under a random-effects model. Certainty of evidence was evaluated using the GRADE framework.

**Results:**

Four RCTs were included, with 77 participants allocated to HILT combined with exercise and 92 to control groups. The overall RoB was assessed as high across all included trials, primarily driven by concerns related to outcome measurement and selective reporting. All HILT protocols were delivered alongside therapeutic exercise (strengthening and/or stretching), and no included trial evaluated HILT as a standalone intervention. HILT resulted in a statistically significant reduction in pain at post-treatment (SMD = − 0.66; 95% CI − 0.98 to − 0.35; *p* < 0.01) and at follow-up (SMD = − 1.08; 95% CI − 1.89 to − 0.26; *p* < 0.01); however, the certainty of evidence was rated as very low due to high RoB across included trials, substantial heterogeneity (I^2^ > 50%), and imprecision related to small sample sizes. No significant between-group differences were observed for functional outcomes (Kujala scale), for which the certainty of evidence was also very low due to similar methodological limitations.

**Conclusion:**

HILT combined with therapeutic exercise may reduce pain in individuals with PFP; however, its independent effect cannot be determined. The very low certainty of the evidence limits confidence in these findings, and improvements in functional outcomes remain unclear. High-quality randomized controlled trials are required to clarify the role of HILT in PFP rehabilitation and to determine its potential contribution within multimodal treatment approaches.

**Trial registration:**

CRD42025631248 (PROSPERO) (December 24, 2024).

**Supplementary Information:**

The online version contains supplementary material available at 10.1186/s12998-026-00653-z.

## Introduction

Globally, an estimated 1.71 billion people are affected by musculoskeletal disorders, with knee pain representing the third most prevalent condition after low back pain and neck pain [1, 2]. Knee pain affects approximately 25% of adults, and its prevalence has increased markedly over the past two decades, accounting for nearly 4 million primary care visits annually [3]. The most common causes of knee pain include osteoarthritis, ligamentous injuries, and patellofemoral pain (PFP), also known as runner’s knee [3]. Among these conditions, PFP is one of the most frequent sources of anterior knee pain in outpatient and rehabilitation settings, particularly among physically active individuals and athletes [4, 5]. Epidemiological studies estimate that PFP affects approximately 3–6% of the general population and occurs more frequently in women [4]. Due to its multifactorial nature and symptom provocation during functional activities such as running, stair negotiation, and squatting, PFP represents a frequent clinical challenge requiring comprehensive assessment and individualized management strategies [5].

Clinically, PFP is characterized by retropatellar or peripatellar pain and is especially common in physically active young women, typically between the second and third decades of life [3]. Its etiology is multifactorial, involving overuse, biomechanical alterations, and muscle dysfunction [5]. Biomechanical contributors such as femoral anteversion, patellar malalignment, increased Q angle, and altered tibial rotation may disrupt the alignment of the extensor mechanism and contribute to symptom development [3–5]. In addition to these physical contributors, growing evidence suggests that psychological factors—such as fear-avoidance behaviors, maladaptive pain beliefs, and reduced self-efficacy—may influence pain persistence and functional limitations in individuals with PFP, supporting a biopsychosocial perspective of the condition [6]. Symptoms are often aggravated by weight-bearing activities and may restrict participation in physical, sports, and occupational activities, frequently leading to persistent or recurrent symptoms [3, 5, 7].

Medical management of PFP may include nonsteroidal anti-inflammatory drugs (NSAIDs), although their benefits are generally modest and short-lived. In selected cases with structural abnormalities, surgical interventions may be considered [5, 8]. Physical therapy, particularly exercise-based interventions, represents the first-line treatment for PFP in both general and athletic populations, as recommended by the 2018 International Patellofemoral Pain Research Retreat Consensus Statement [8]. Strengthening of the quadriceps and hip musculature is commonly prescribed to improve patellar alignment and dynamic knee control [8–11]. Early initiation of physical therapy has been associated with lower healthcare utilization, reduced costs, and a lower risk of recurrent knee pain compared with delayed intervention [6, 10–13]. Within this context, physical agents are commonly used as adjuncts in multimodal rehabilitation programs combining exercise, education, and activity modification. Among these modalities, radiofrequency diathermy, radial shockwave therapy, and neuromuscular electrical stimulation (NMES) combined with exercise seem to improve pain, function, and quadriceps strength, as suggested by randomized trials and recent evidence from a systematic review evaluating adjunct interventions for patellofemoral pain [13–15].

Laser-based therapies have increasingly emerged as adjunctive modalities in musculoskeletal rehabilitation and are applied by various health professionals, including physical therapists and chiropractors. Low-level laser therapy (LLLT) has demonstrated analgesic and functional benefits in several musculoskeletal conditions, including PFP [16, 17]. Although both LLLT and high-intensity laser therapy (HILT) exert photobiomodulatory effects that modulate inflammatory mediators and nociceptive activity, they differ in their thermal profiles. HILT operates at higher power outputs (> 0.5 W), producing thermal effects in addition to photobiomodulation that may enhance analgesic responses and lead to therapeutic effects distinct from those of LLLT [18–20]. These characteristics highlight the need to investigate whether HILT provides meaningful therapeutic effects in PFP.

Within this pathophysiological framework, the biological effects of HILT may be mechanistically relevant for PFP. Through combined photobiomodulatory and thermal effects, HILT may modulate inflammatory mediators, enhance local microcirculation, and influence nociceptive signaling—mechanisms potentially relevant to pain persistence, peripatellar sensitization, and altered tissue homeostasis observed in PFP [18, 20–22]. However, given the complex, load-dependent, and multifactorial nature of PFP [6, 9, 12], the presence of plausible biological mechanisms does not necessarily guarantee clinically meaningful effects, highlighting the need for condition-specific evaluation of HILT in this population.

The therapeutic potential of HILT in PFP remains largely unexplored. Although evidence from other musculoskeletal conditions—particularly knee osteoarthritis—consistently supports its analgesic and functional effects [19–24], it is uncertain whether these benefits translate to PFP, a condition with distinct biomechanical and pathophysiological features. Importantly, no systematic review has yet examined the randomized controlled trials (RCTs) evaluating the effects of HILT specifically in PFP. This lack of synthesized evidence limits clinicians’ ability to determine the potential therapeutic value of HILT for this population. To address this gap, the primary aim of this systematic review was to evaluate the effects of high-intensity laser therapy (HILT) on pain intensity in individuals with patellofemoral pain. Secondary aims were to assess its effects on functional outcomes and disability.

## Methods

### Study design

This study is a systematic review and meta-analysis conducted in accordance with the Preferred Reporting Items for Systematic Reviews and Meta-Analyses (PRISMA 2020) guidelines [25]. The protocol was prospectively registered in the International Prospective Register of Systematic Reviews (PROSPERO; CRD42025631248; December 24, 2024).

### Selection criteria

Eligibility criteria were structured according to the PICOS framework (Population, Intervention, Comparison, Outcomes, and Study design) [26]. Participants of both sexes with a clinical diagnosis of PFP were considered eligible. PFP was defined as anterior knee pain (retropatellar or peripatellar) reproduced during at least two common functional activities (e.g., squatting, stair climbing/descending, running, or prolonged sitting), with symptoms lasting ≥ 3 months and no evidence of other knee pathologies (such as osteoarthritis, ligamentous, or meniscal injury) [6, 9, 12]. This definition is consistent with internationally accepted diagnostic criteria, including the 2018 International Patellofemoral Pain Research Retreat consensus statement [9]. Interventions of interest included HILT (Class IV or high-power laser), defined as laser therapy delivered with a power output > 0.5 W, in accordance with current technical and clinical guidelines [20, 21]. HILT could be administered either as a standalone modality or as an adjunct to other physiotherapy interventions (e.g., exercise-based rehabilitation). Comparator conditions included exercise-based rehabilitation, other physical therapy approaches, electrophysical agents, or sham-controlled protocols. No restrictions were applied regarding the comparator intervention, allowing the effects of HILT to be evaluated across different rehabilitation approaches. When HILT was delivered as an adjunct to physical therapy intervention, comparator groups received the same co-intervention (e.g., exercise-based rehabilitation), with or without an alternative electrophysical agent. The primary outcome was pain intensity, assessed using validated scales such as the Visual Analogue Scale (VAS) or the Numerical Pain Rating Scale (NPRS). Secondary outcomes included function, measured with instruments such as the Kujala Scale, the functional subscales of the Western Ontario and McMaster Universities Osteoarthritis Index (WOMAC), or other comparable functional measures relevant to PFP. No language restrictions were applied during study selection.

Exclusion criteria included studies without a control group, studies involving PFP associated with other musculoskeletal or neurological conditions of the knee or lower limb, and studies with insufficient data or methodological detail to allow data extraction.

### Literature review

An electronic search for RCTs on HILT in PFP was conducted in PubMed, Scopus, Web of Science, the Cumulative Index to Nursing and Allied Health Literature (CINAHL), MEDLINE, ScienceDirect, the Cochrane Library, and PEDro up to May 1, 2026. Although EMBASE was originally specified in the PROSPERO protocol, it was not included in the final updated search due to institutional access limitations at the time the search was performed. Additional studies were identified through Google Scholar to capture potentially relevant grey literature, including theses, conference proceedings, preprints, and other non-indexed RCTs, in accordance with recommended practices for comprehensive systematic review searches [25, 26]. To enhance reproducibility, the first 200 records retrieved for each Google Scholar query were screened. In addition, the reference lists of included trials and relevant systematic reviews were manually screened to identify additional eligible studies [25].

The search strategy combined Medical Subject Headings (MeSH) and free-text terms related to the intervention, using the Boolean operators “OR” and “AND”. The keywords included “Lasers,” “Laser Therapy,” "Photobiomodulation Therapy," “High-Intensity Laser Therapy,” “Class IV laser,” “Musculoskeletal Pain,” “Musculoskeletal Diseases,” “Knee Joint,” “Patellofemoral Pain Syndrome,” “Patellofemoral Joint,” and “Pain Management.” These terms were integrated into the following search algorithm: *("Lasers" OR "Laser Therapy" OR "High-Intensity Laser Therapy" OR “High-power laser” OR "Class IV laser")* AND *("Musculoskeletal Pain" OR "Musculoskeletal Diseases" OR "Knee Joint" OR "Patellofemoral Pain Syndrome" OR "Patellofemoral Joint" OR "Pain Management").* No filters were applied to ensure maximal coverage and retrieval of relevant studies.

Titles and abstracts were independently screened by two reviewers (HDB and NAP) using the Rayyan® web tool [28], with disagreements resolved by a third reviewer (REL). Data extraction covered study authorship, country, participant characteristics, interventions, outcome measures, funding sources, and HILT technical parameters.

### Methodological quality

Methodological quality was assessed using the Physiotherapy Evidence Database (PEDro) scale [29]. The PEDro database was initially consulted to obtain a descriptive overview of the methodological quality and trial design characteristics.

### Risk of bias

Risk of bias (RoB) was evaluated using the Cochrane Collaboration’s Risk of Bias 2 (RoB 2) tool [30], with overall judgments assigned according to the Cochrane algorithm. This assessment covered five domains: the randomization process, deviations from intended interventions, missing outcome data, measurement of the outcome, and selection of the reported result. A study was classified as having an overall high RoB if at least one domain was rated as high risk. An overall high RoB was assigned when at least one domain was rated as high risk or when multiple domains raised some concerns that were judged to substantially undermine confidence in the results, in accordance with RoB 2 guidance [30]. Three reviewers (HDB, NAP, and REL) independently assessed all studies using the RoB 2 tool. Inter-rater agreement among the three evaluators was quantified using Fleiss’ kappa statistic [31] and interpreted according to the Landis and Koch classification as follows: poor (< 0.00), slight (0.00–0.20), fair (0.21–0.40), moderate (0.41–0.60), substantial (0.61–0.80), and almost perfect (0.81–1.00) [32].

### Statistical analysis

For the quantitative synthesis, comparisons were grouped according to the intervention received by the experimental and control groups. Trials comparing HILT combined with exercise-based rehabilitation versus non-HILT physiotherapy interventions (e.g., exercise alone, exercise combined with other electrophysical agents, or exercise plus placebo HILT) were pooled. Outcomes were synthesized according to the timepoint reported in the primary studies.

Meta-analyses were conducted for pain intensity and disability/functional outcomes when at least two eligible studies reported these variables [33]. Standardized mean differences (SMDs) with 95% confidence intervals (CIs) were calculated using the inverse-variance method, which applies greater weight to studies with lower variance [34]. When an outcome was assessed using different instruments across studies, the most frequently reported or widely used scale was prioritized for quantitative synthesis to ensure clinical and construct homogeneity. In three-arm trials, the shared control group was split into subgroups with proportionally adjusted sample sizes, following Cochrane Handbook recommendations, to enable independent pairwise comparisons while avoiding double-counting of participants and overestimation of precision [35]. For pain intensity, negative SMD values indicate greater improvement in the HILT group compared with control interventions, whereas for functional outcomes, positive SMD values reflect better functional status in participants receiving HILT. Two assessment points were considered for meta-analysis: post-treatment (end of treatment) and follow-up, defined as assessments conducted between 4 and 12 weeks after the intervention.

Heterogeneity was assessed with the chi-squared test and quantified using the I^2^ statistic to estimate statistical heterogeneity across studies, with values > 50% indicating substantial heterogeneity [36, 37]. Depending on the level of statistical heterogeneity (I^2^), pooled effects were estimated using either the Mantel–Haenszel fixed-effects model or the DerSimonian–Laird random-effects model [35]. Given the anticipated clinical and methodological heterogeneity across studies—particularly regarding comparator interventions, outcome measures, and HILT parameters—a random-effects model was considered the most appropriate primary analytical approach.

SMDs were interpreted according to Cohen’s d, categorized as small (0.2–0.5), moderate (0.5–0.8), or large (≥ 0.8) effects [37, 38]. Sensitivity analyses were planned a priori to assess the robustness of the pooled estimates in the presence of anticipated clinical and methodological heterogeneity across studies, including the exclusion of trials judged to be at higher RoB. Subgroup analyses were not planned because, given the expected limited number of eligible trials and small sample sizes, such analyses would likely have resulted in underpowered and unreliable estimates [38].

A graphical and statistical assessment of publication bias was planned using funnel plots and Egger’s regression test when ≥ 10 RCTs were available, as recommended by the Cochrane Handbook [35]. All analyses were performed using Review Manager (RevMan) version 5.4.1, provided by the Cochrane Collaboration.

### Clinical relevance

Clinical relevance was evaluated using effect size thresholds widely applied in the behavioral and health sciences [36]. Small clinical importance was defined as an SMD ≤ 0.5; medium clinical importance as an SMD between 0.5 and 0.8; and large clinical importance as an SMD ≥ 0.8 [37, 38]. These thresholds originate from established behavioral science conventions and are consistent with methodological recommendations within the Cochrane framework [35–37].

### Certainty of the evidence

The certainty of the evidence was assessed using the GRADE (Grading of Recommendations Assessment, Development and Evaluation) approach [39]. The certainty assessment considered five domains: risk of bias, inconsistency, indirectness, imprecision, and publication bias [38, 39]. Because all included studies were randomized controlled trials, the evidence initially started at a high level and was downgraded when concerns were identified across these domains. Outcome importance was defined a priori according to GRADE guidance, based on relevance for clinical decision-making. In this review, pain intensity and functional outcomes were classified as critical outcomes because they represent the most clinically relevant patient-centered outcomes in individuals with patellofemoral pain and are commonly used to evaluate treatment effectiveness. The certainty of evidence ranged from high to very low. A Summary of Findings table was generated using GRADEpro GDT (McMaster University, Hamilton, Canada). (https://gdt.gradepro.org/app/).

## Results

### Database search

**Fig. 1:**
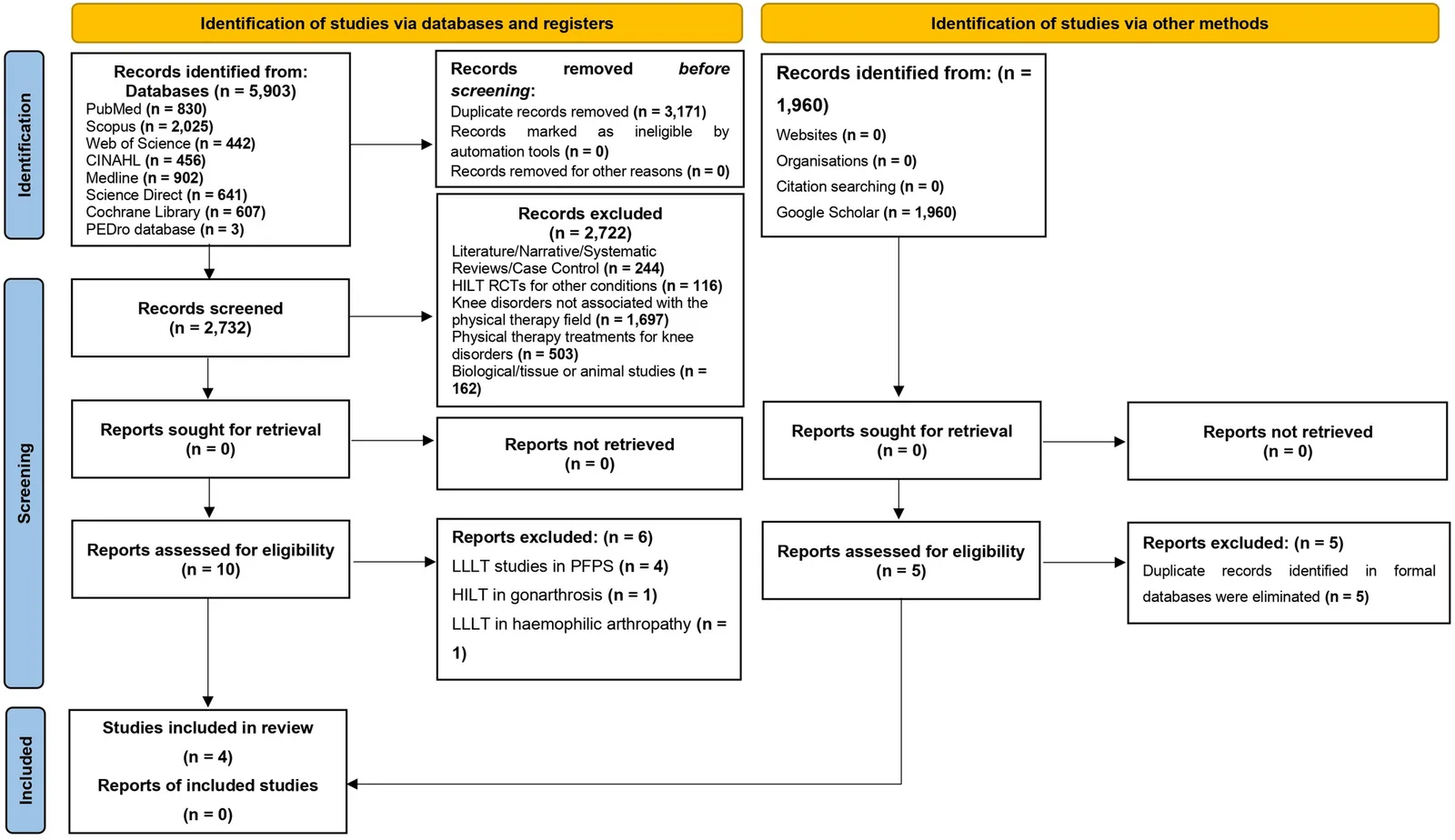
PRISMA flow diagram

The search yielded 5,903 records (last updated May 1, 2026), with 1,960 additional studies identified through other sources. After removal of duplicates, 2,732 unique records remained. Screening of titles and abstracts identified 10 studies, of which 6 were excluded (LLLT in patellofemoral pain syndrome, n = 4; HILT in gonarthrosis, n = 1; LLLT in chondromalacia patellae, n = 1). An additional search in Google Scholar identified five records, all of which were duplicates. Four studies met the eligibility criteria and were included [40–43]. The PRISMA flow diagram is presented in Fig. 1. The complete search strategy is detailed in Supplementary Table 1. A comprehensive list of excluded studies, together with explicit reasons for exclusion, is provided in Supplementary Table 2.

### Methodological quality of included RCTs

The PEDro scores of the included RCTs ranged from 4 to 8 out of 10, indicating low to moderate methodological quality [29]. The lowest-scoring PEDro items were related to allocation concealment and blinding procedures (participants, therapists, and outcome assessors). The detailed assessment of methodological quality using the PEDro scale is presented in Supplementary Table 3.

### Risk of bias of included RCTs

**Fig. 2:**
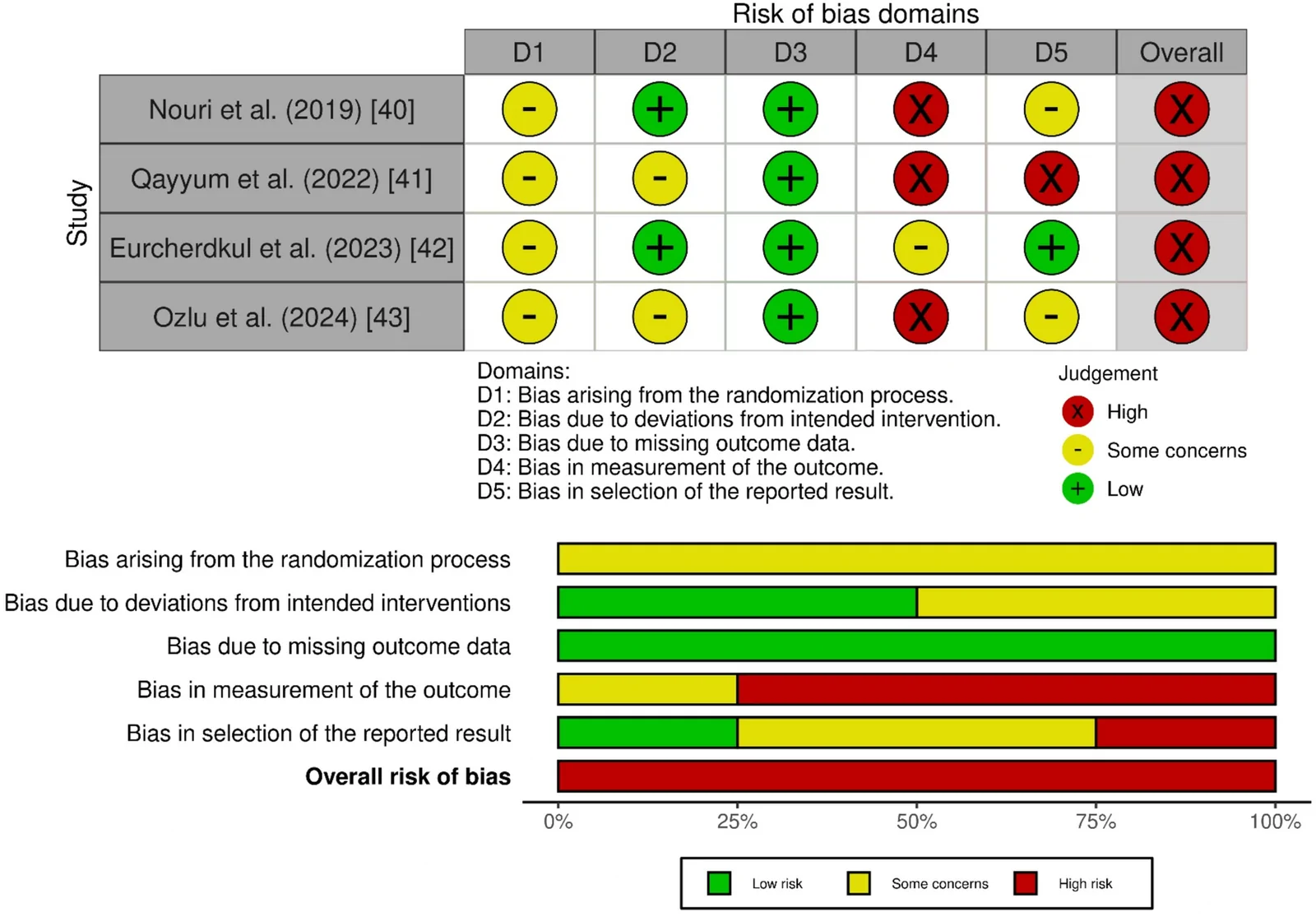
Risk of bias assessment of included trials

Figure 2 presents the RoB 2 assessment of the included studies [30]. Inter-rater agreement among the three reviewers was high, with a mean Fleiss’ kappa of 0.82 (range: 0.69–1.00), indicating substantial to almost perfect agreement according to the Landis and Koch classification [31, 32]. Overall, all included trials were judged to have a high RoB, primarily driven by concerns related to outcome measurement, deviations from intended interventions, and selective reporting. Regarding individual domains, all trials presented some concerns in the randomization process, mainly because allocation concealment was not clearly documented. Missing outcome data was judged to be at low risk across studies. Deviations from intended interventions generated additional concerns in 50% of the studies [41, 43]. Outcome measurement was the most frequent source of bias, largely due to the absence of blinding of treating clinicians or outcome assessors, with 75% of trials rated as high risk in this domain [40, 41, 43].

### Characteristics of the included RCTs

Table 1 summarizes the characteristics of the included RCTs. PEDro scores ranged from 4 to 8, with a mean score of 5.8 (SD 1.7). Consistent with standard reporting practices, scores are presented descriptively without qualitative labels. Half of the trials scored ≥ 6 on the PEDro scale [40, 42]. Across studies, the most frequently unmet criteria were allocation concealment (item 3) and blinding of participants or outcome assessors (item 6), whereas criteria related to statistical reporting—between-group comparisons (item 10) and point estimates with variability measures (item 11)—were consistently fulfilled.

**Table 1:** Characteristics of included studies

First author (year) Country	PEDro score	Participants (n) mean age (SD)	Selection criteria	Groups (n)	Sessions	Outcomes (instrument)	Assessment instances	Baseline pain and functional status (mean and SD)	Results after treatment	Study conclusion	Adverse effects	Source of funding
Nouri et al. [40] Iran	6/10	40 (M 12, F 28) 33.4 (5.0) years Dropout 4 (2 per group)	Inclusion criteria: Clinical diagnosis of PFPS Anterior knee or retropatellar pain Symptoms lasting ≥ 3 months At least two positive PFPS clinical tests (Shrug, Grind, Perkins tests) Normal knee X-ray (AP, lateral, patellar views) Age 15–40 years Exclusion criteria: History of knee surgery or recent trauma Knee OA or deformity BMI > 30 kg/m^2^ Systemic diseases (e.g., diabetes, collagen vascular disorders) Malignancy, infection Pregnancy or breastfeeding	EG (20): HILT + EX (strengthening exercise) CG (20): HILT placebo + EX (strengthening exercise)	2 s/week for 2 weeks	(A) PI (VAS) (B) PI (Pain WOMAC) (C) Disability (Function domain of WOMAC) (D) Stiffness (Stiffness domain of WOMAC) (E) Function (Kujala scale)	T0: baseline T1: post-treatment (4 weeks) T2: follow-up (12 weeks)	(A) VAS: EG = 6.5 (NS); CG = 5.5 (NS) (B) WOMAC Pain: EG = 9.0 (NS); CG = 8.0 (NS) (C) WOMAC Function: EG = 22.0 (NS); CG = 21.0 (NS) (D) WOMAC Stiffness: EG = 2.5 (NS); CG = 2.5 (NS) (E) Kujala scale: EG = 76.0 (NS); CG = 82.0 (NS)	EG: ↓PI*, ↓Disability*, ↓Stiffness*, and ↑Function CG: ↓PI*, ↓Disability*, ↓Stiffness*, and ↑Function EG < CG for PI*, PI*, Disability*, Stiffness* EG > CG for Function	Short-term HILT combined with an appropriate EX regimen significantly reduces pain in patients with PFPS. However, its effectiveness for improving function has not been demonstrated	None	NS
Qayyum et al. [41] Pakistan	4/10	66 (M 35, F 31) 27.7 (6.7) years Dropout 0	Inclusion criteria: Diagnosed PFPS Anterior, retro-patellar, or peri-patellar knee pain Pain duration > 3 months Pain aggravated by functional activities Gradual/insidious onset Pain on patellar palpation, step-down test, or squat Exclusion criteria: Knee osteoarthritis Meniscal injury or joint effusion Patellar subluxation/dislocation Intra-articular pathology or bursitis Prior knee surgery or recent trauma Neurological disorders affecting gait Previous physiotherapy in the last 3 months	EG (33): HILT + EX (stretching and strengthening exercise) + patellar mobilization CG (33): EX (stretching and strengthening exercise) + patellar mobilization	2 s/week for 4 weeks	(A) PI (VAS)	T0: baseline T1: during treatment (2 weeks) T2: post-treatment (4 weeks) T3: follow-up (4 weeks)	(A) VAS: EG = 7.8 (1.0); CG = 7.8 (0.9)	EG: ↓PI* CG: ↓PI* EG < CG for PI*	Combining routine physiotherapy with HILT significantly reduces pain intensity and improves functional disability in PFPS patients	None	NS
Eurcherdkul et al. [42] Thailand	8/10	18 (M 5, F 13) 35.4 (8.2) years Dropout 0	Inclusion criteria: Adults aged 20–50 years Anterior knee pain for ≥ 3 months Pain provoked by ≥ 2 activities Pain score ≥ 3 on VAS Positive patellar grind test Exclusion criteria: History of knee surgery Recent knee trauma or infection Knee OA Use of corticosteroids or anti-inflammatory drugs in the past 6 months Severe neurological or cardiovascular disorders Contraindications to laser therapy	EG (9): HILT + EX (strengthening exercise) CG (9): HILT placebo + EX (strengthening exercise)	2-3 s/week for 3 weeks	(A) PI (VAS) (B) Function (Kujala scale) (C) QoL (SF-36)	T0: baseline T1: post-treatment (3 weeks) T2: follow-up (6 weeks) T3: follow-up (12 weeks)	(A) VAS: EG = 6.0 (2.1); CG = 5.8 (2.8) (B) Kujala scale: EG = 73 (15.5); CG = 75 (16) (C) SF-36: EG = 599.6 (121.0); CG = 578.8 (151.9)	EG: ↓PI, ↑Function, and ↑ QoL CG: ↓PI, ↑Function, and ↑ QoL EG = CG for ↓PI, ↑Function, and ↑ QoL	The efficacy of HILT combined with EX was not significantly different from EX alone in terms of pain reduction, function improvement, and increase in QoL	None	NS
Ozlu et al. [43] Turkey	5/10	49 (M 21, 24 F) 33.8 (6.7) years Dropout 4	Inclusion criteria: Clinical diagnosis of unilateral PFPS confirmed by a physician Anterior knee pain (retropatellar or peripatellar) Pain lasting > 3 months Pain reproduced during ≥ 2 functional activities Positive patellar compression and grind tests Age 25–45 years Exclusion criteria: History of knee trauma or surgery Knee OA on X-ray Other joint diseases Neurological disorders affecting gait Pregnancy	EG (15): HILT + EX (stretching and strengthening exercise) CG1 (15): US + TENS + EX (stretching and strengthening exercise) CG2 (15): US + ITFC + EX (stretching and strengthening exercise)	5 s/week for 2 weeks Exercises were maintained after treatment for 12 weeks	(A) PI (VAS) (B) PPT (ALG) (C) ROM (GNM) (D) Knee strength (DNM) (E) Knee valgus (Q angle) (F) Function (Kujala scale) (G) Function (LEFS) (H) Disability (TUG)	T0: baseline T1: post-treatment (2 weeks) T2: follow-up (12 weeks)	(A) VAS: EG = 6.8 (1.7); CG = 6.9 (2.1) (B) Kujala scale: EG = 64.4 (14.5); CG = 66.4 (10.1) (C) LEFS: EG = 50.7 (15.0); CG = 49.3 (14.4) (D) TUG: EG = 8.2 (2.0); CG = 8.5 (1.4)	EG: ↓PI*, ↑PPT*, ↑ROM*, ↑Knee strength*, ↓Knee valgus*, ↑Function* (Kujala scale), ↑Function* (LEFS), and ↓Disability* (TUG) CG1: ↓PI*, ↑PPT*, ↑ROM*, ↑Knee strength*, ↓Knee valgus*, ↑Function* (Kujala scale), ↑Function* (LEFS), and ↓Disability* (TUG) CG2: ↓PI*, ↑PPT*, ↑ROM*, ↑Knee strength*, ↓Knee valgus*, ↑Function* (Kujala scale), ↑Function* (LEFS), and ↓Disability* (TUG) EG < CG2 < CG1 for PI* and Disability* (TUG) EG = CG1 = CG2 for PPT, Quadriceps strength, and Knee valgus EG > CG1 > CG2 for ROM* and Hamstring strength* EG > CG2 > CG1 for Function* (Kujala scale) and Function* (LEFS)	HILT is more effective than US plus TENS or US plus ITFC for treating PFPS after 3 months, especially when combined with vastus medialis strengthening exercises	None	Scientific and Technological Research Council of Turkiye (TUBITAK)

ALG, Algometry; CG, Control Group; DNM, Dynamometry; EG, Experimental Group; EX, Exercises; F, Female; GNM, Goniometry; HILT, High Intensity Laser Therapy; ITFC, Interferential Currents; LEFS, Lower Extremity Functional Scale; M, Male; NS, Not Specified; NS, Not specified; OA, Osteoarthritis; PEDro, Physiotherapy Database Evidence; PI, Pain Intensity; PFPS, Patellofemoral Pain Syndrome; PPT, Pressure Pain Threshold; QoL, quality of life; ROM, range of motion; SD, Standard Deviation; TENS, Transcutaneous Electrical Nerve Stimulation; TUG, Time Up and Go; US, Therapeutic Ultrasound; VAS, Visual Analog Scale; WOMAC, Western Ontario McMaster Universities Osteoarthritis Index.*p < 0.05

In total, 173 participants were included across the four trials, comprising 73 men (42.2%) and 96 women (57.8%), with a mean age of 37.3 years (SD 6.7). The studies were conducted between 2019 to 2024 in Iran [40], Pakistan [41], Thailand [42], and Turkey [43]. Two trials reported losses to follow-up, comprising eight participants overall [40–43].

All interventions combined HILT with therapeutic exercise (strengthening and/or stretching). In two trials, the exercise program focused on lower-limb strengthening (quadriceps femoris, hamstrings, abductors, and adductors), with particular emphasis on the quadriceps femoris through isometric exercises and straight leg raises [40, 42]. Two RCTs incorporated strengthening followed by stretching, targeting the hamstrings, calf muscles, and iliotibial band, with one also including patellar mobilization [41, 42].

Regarding comparators, two trials used sham HILT combined with the same strengthening program applied in the intervention group [40, 42], with one of them also adding patellar mobilization [41]. Another trial included two active control arms: therapeutic ultrasound (US) plus stretching exercises combined with transcutaneous electrical nerve stimulation (TENS), or the same protocol replacing TENS with interferential current [42].

The number of treatment sessions ranged from four to ten (mean seven), delivered two to five times per week over a period of two to four weeks. All trials reported post-treatment outcomes and follow-up assessments conducted between 4 and 12 weeks [40–43]. No adverse events related to HILT were reported in any of the included trials.

### Main outcomes

Pain intensity, assessed with the VAS, was reported in all four trials [40–42]. At baseline, participants presented with moderate to high pain intensity (mean VAS ≈ 5.5–7.8), with comparable values between intervention and control groups across studies (Table 1). The WOMAC index was applied in one study to assess pain, disability, and stiffness [40]. Physical function was measured with the Kujala Anterior Knee Pain Scale in three studies [40, 41, 43] and the Lower Extremity Functional Scale (LEFS) in one [43]. Baseline functional status was also comparable between groups, indicating moderate functional impairment (Kujala ≈ 64–82; LEFS ≈ 49–51), with no relevant imbalances at study entry (Table 1) [40–43]. Across the included RCTs, consistent reductions in pain were reported when HILT was combined with exercise compared with control interventions [40–43]. Improvements in functional outcomes (Kujala and WOMAC scores) were reported in two studies [41, 43]. Overall, evidence regarding additional benefits on functional outcomes remains limited, although one trial reported greater improvements with HILT compared with ultrasound combined with TENS or interferential current [43].

### Additional outcomes reported in the included studies

Additional outcomes were also extracted and summarized descriptively. Quality of life was assessed using the SF-36 in a single study [42]. Less frequently reported outcomes included pressure pain threshold measured by algometry, knee range of motion assessed by goniometry, quadriceps and hamstring strength evaluated with dynamometry, Q-angle measurement for knee valgus alignment, and the Timed Up and Go test for disability [41].

### HILT parameters

Table 2 presents the characteristics of the lasers applied in the included trials. All trials applied a wavelength of 1064 nm [40–43]. Continuous emission was the most common mode [42, 43], whereas pulsed [40] and combined continuous/pulsed modes [41] were less frequently reported. Peak output power varied substantially, ranging from 7–12 W [40, 41, 43] to 3000 W in one [42]. Mean power was more consistent, with an average of 8.6 W (SD 3.2) across three trials [40–43]. Spot size showed high heterogeneity, with two studies reporting 0.8 cm^2^ [40, 41], one not specifying [42], and one applying 25 cm^2^ [43]. Energy density values ranged from 1.53 J/cm^2^ [42] to 150 J/cm^2^ [43], with 120 J/cm^2^ most frequently reported [40, 41] (mean 100.6 J/cm^2^, SD 65.8). Total energy delivered averaged 1200 J, spanning 300 J [40, 43] to 3000 J [40]. Treatment duration clustered between 120 and 360 s, with a mean of 221 s (SD 100) [40–43].

**Table 2:** Technical specifications of HILT protocols in the included studies

Study	Laser model	Laser medium	Emission mode (continuous/pulsed)	Frequency (Hz)	Duty cycle (%)	Wavelength (nm)	Nominal Peak Power (W)	Mean power (W)*	Fluency (J/cm^2^)	Spot size (cm^2^)	Treatment time (s)	Treatment position	Protocol
Nouri [40]	BTL-6000	Nd:YAG	Pulsed	NS	25%	1064	12	4	120	0.8	120	Supine position, knee in full extension	Scanning application around patellar margins
Qayyum [41]	NS	NS	Continuous/pulsed	NS	NS	1064	NS	10	120	0.8	120	Supine position, knee in full extension	Scanning application around patellar margins
Eucherdkul [42]	Hilterapia® HIRO TT® (ASA laser)	Nd:YAG	Pulsed	20 Hz	NS	1064	~ 3,000	10.5	1.53	NS	285	Supine position, knee flexion 30°	Point application, 5 sites around the knee
Ozlu [43]	iLux Laser (Mectronic Medicale)	Nd:YAG	Continuous	25 Hz	NS	1064	7–10	NS	12–150	25	240–360	Supine position, knee in full extension	Direct contact

^*^Mean power (W) represents the power effectively delivered to the patient

cm^2^, square centimeters; Hz, hertz; J/cm^2^, joules per square centimeter; Nd:YAG, neodymium-doped yttrium aluminum garnet; NS, not specified; s, seconds; W, watt; nm, nanometer

### Quantitative synthesis: meta-analysis

#### Pain intensity

**Fig. 3:**
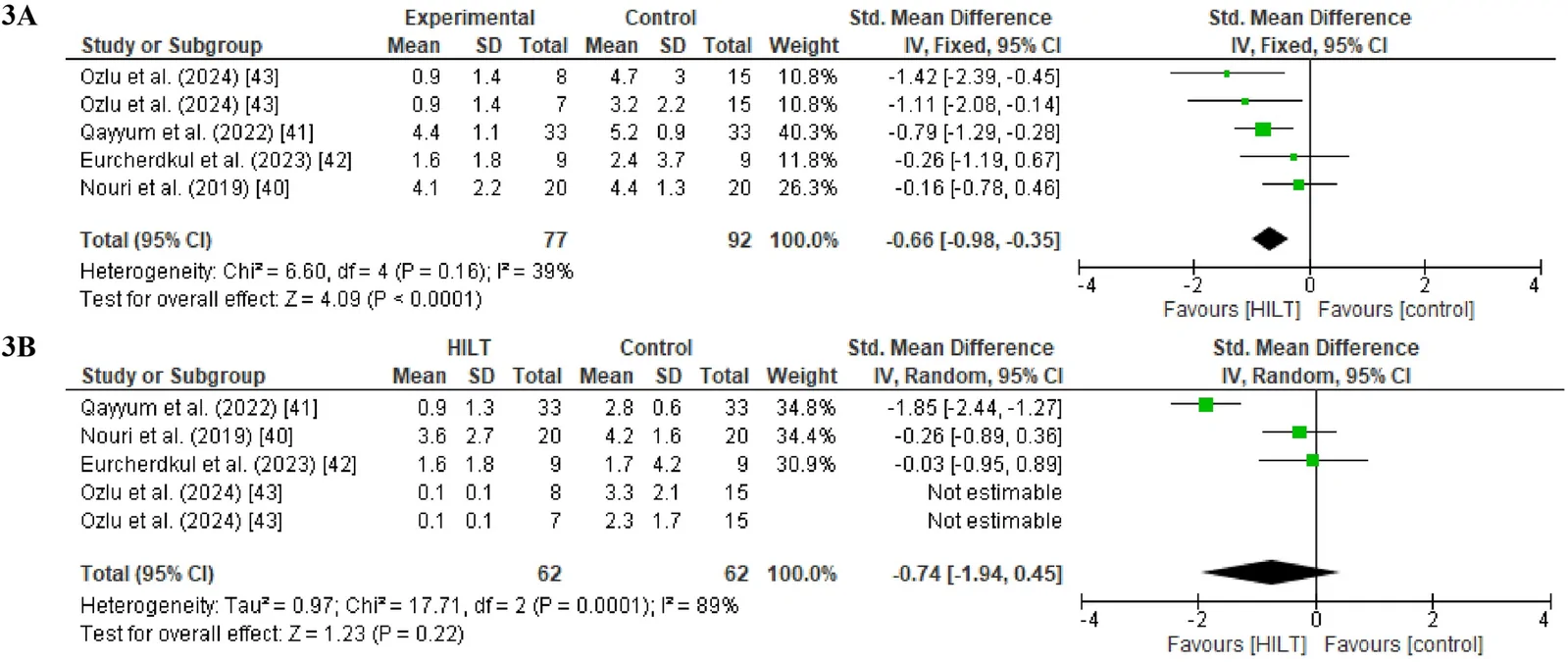
Forest plot of pain intensity at the end of treatment (**3A**) and at follow-up (**3B**) (VAS): comparison of HILT with physical therapy interventions, including HILT placebo plus therapeutic exercise (strengthening and/or stretching), patellar mobilization, therapeutic ultrasound, transcutaneous electrical nerve stimulation (TENS), and interferential current therapy

Four RCTs [40–43] (n = 169 participants) comparing HILT combined with exercise with control physical therapy interventions were included in the meta-analysis for pain intensity. At post-treatment (Fig. 3A), the pooled analysis showed a statistically significant reduction in pain favoring HILT (SMD = − 0.66; 95% CI − 0.98 to − 0.35). At follow-up (4–12 weeks) (Fig. 3B), subgroup analysis demonstrated a larger and statistically significant effect (SMD = − 1.08; 95% CI − 1.89 to − 0.26). According to the GRADE framework, pain intensity was considered a critical outcome and the certainty of the evidence was rated as very low [39].

The certainty of the evidence for pain intensity was rated as very low due to serious concerns related to RoB, inconsistency, and imprecision (Table 3) [40–43]. Inconsistency was downgraded because of substantial statistical heterogeneity across studies (I^2^ > 50%). Imprecision was downgraded because the pooled sample size did not reach the predefined optimal information size threshold of 400 participants, and confidence intervals were wide [39].

**Table 3:** Summary of findings and certainty of evidence (GRADE) for statistically significant outcomes

Certainty assessment							№ of patients		Absolute	Certainty	Importance^f^
Studies	Study design	Risk of bias	Inconsistency	Indirectness	Imprecision	Publication bias	High-intensity laser therapy	Conventional physical therapy	(95% CI)		
*Pain intensity after treatment (VAS)*											
4	RCTs [40–43]	Serious^a^ (− 1)	Serious^b^ (− 1)	Not serious^c^ (0)	Serious^d^ (− 1)	None^e^	77	77	**SMD = -0.66**	⨁◯◯◯ Very Low	Critical
									(− 0.98 to − 0.35)		
*Pain intensity at follow-up between 4 and 12 weeks (VAS)*											
4	RCTs [40–43]	Serious^a^ (− 1)	Serious^b^ (− 1)	Not serious^c^ (0)	Serious^d^ (− 1)	None^e^	77	77	**SMD = -1.08** (− 1.89 to − 0.26)	⨁◯◯◯ Very Low	Critical
*Function after treatment (Kujala Scale)*											
3	RCTs [40, 41, 43]	Serious^a^ (− 1)	Serious^b^ (− 1)	Not serious^c^ (0)	Serious^d^ (− 1)	None^e^	44	59	**SMD = 0.57**	⨁◯◯◯ Very Low	Critical
									(− 0.62 to 1.77)		
*Function at follow-up 4 to 12 weeks (LEFS and Kujala Scale)*											
3	RCTs [40, 41, 43]	Serious^a^ (− 1)	Serious^b^ (− 1)	Not serious^c^ (0)	Serious^d^ (− 1)	None^e^	44	59	**SMD = 0.22**	⨁◯◯◯ Very Low	Critical
									(− 0.61 to 1.05)		

CI, confidence interval; RCT, randomized controlled trial; SMD, standardized mean difference; VAS, visual analog scale

^a^The high risk of bias was mainly associated with outcome measurement and with selective reporting of results, as assessed using the Cochrane Risk of Bias 2 tool

^b^Statistical heterogeneity was substantial, as indicated by the I^2^ statistic (I^2^ > 50%)

^c^Due to the direct comparison of interventions and outcomes specifically related to the study's issue, it was determined that the indirect evidence held little significance

^d^The imprecision was evaluated considering the width of the confidence interval (CI) for the pooled mean difference and the crossing of the no-effect line in the meta-analysis, and the sample size (n < 400)

^e^The meta-analysis included at least three studies

^f^Outcome importance (not important, important, or critical) was defined a priori according to GRADE guidance, based on relevance for clinical decision-making [39, 44]

Based on the predefined effect size thresholds (SMD 0.5–0.8 indicating moderate and ≥ 0.8 indicating large clinical importance), the post-treatment effect showed moderate clinical relevance, whereas the follow-up effect was of large potential clinical relevance [38].

#### Pain intensity sensitivity analysis

**Fig. 4:**
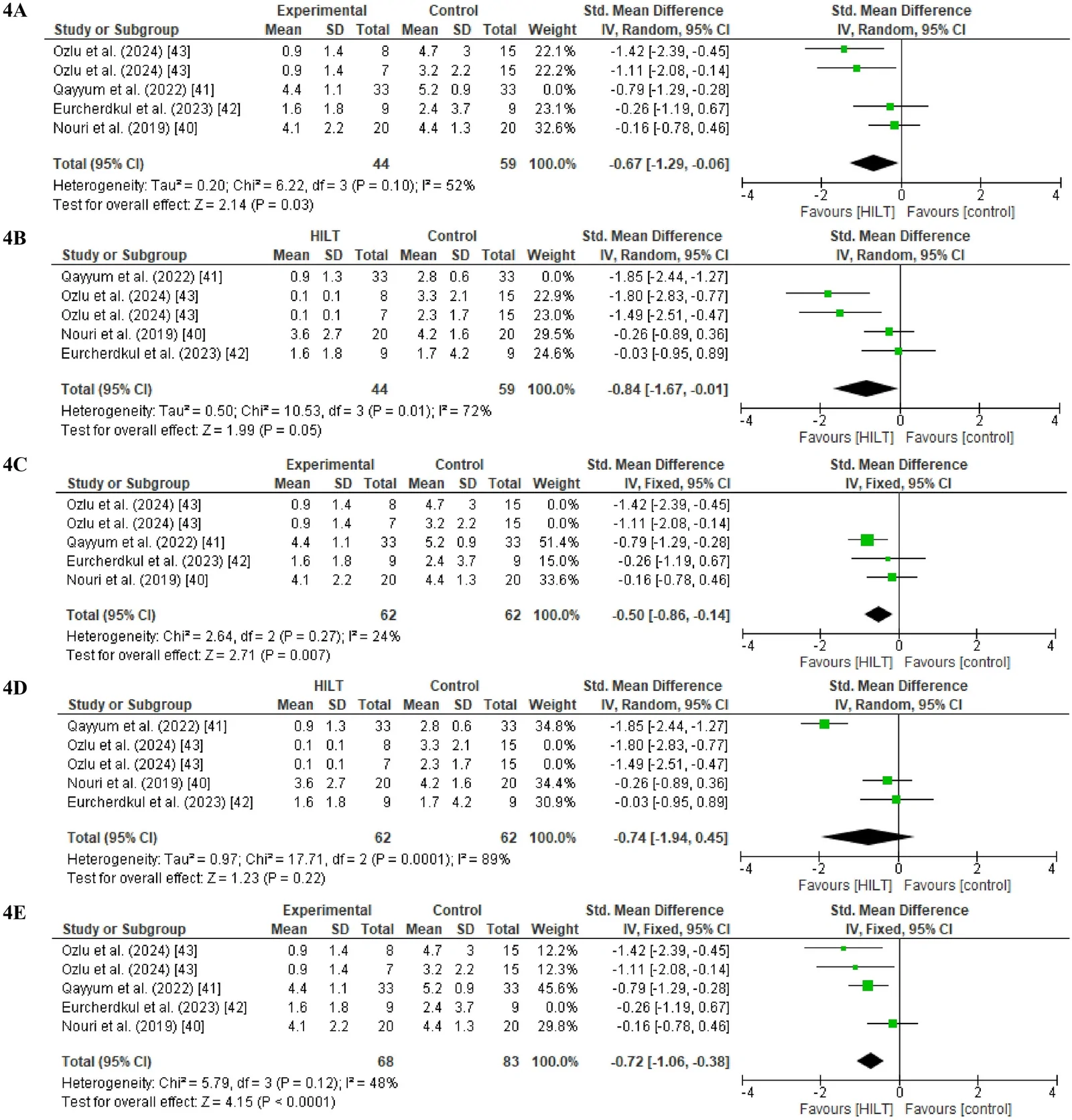
Forest plots of pain intensity assessed using the VAS at post-treatment and at follow-up. Sensitivity analyses were performed to evaluate the robustness of the pooled estimates by excluding individual studies. Specifically, analyses excluding Qayyum et al. [41] are presented in panels 4A and 4B, exclusion of Ozlu et al. [43] is shown in panel 4C, and exclusion of Eurcherdkul et al. [42] is presented in panel 4E. The analyses compare HILT with other physical therapy interventions, including HILT placebo combined with therapeutic exercise (strengthening and/or stretching), patellar mobilization, therapeutic ultrasound, transcutaneous electrical nerve stimulation (TENS), and interferential current therapy

Sensitivity analyses were conducted by sequentially excluding the trials with the highest RoB according to the RoB 2 assessment (Quayyum et al. [41] and Ozlu et al. [43]) for pain intensity outcomes at post-treatment (Fig. 4A and C) and at follow-up (4–12 weeks) (Fig. 4B and D).

At post-treatment, exclusion of Quayyum et al. [41] yielded a statistically significant reduction in pain favoring HILT with a moderate effect size (SMD = − 0.67; 95% CI − 1.29 to − 0.06; EG = 44, CG = 59). Similarly, exclusion of Ozlu et al. [43] resulted in a statistically significant effect favoring HILT (SMD = − 0.50; 95% CI − 0.86 to − 0.14; EG = 44, CG = 59).

For follow-up outcomes (4–12 weeks), sensitivity analyses excluding either Quayyum et al. [41] or Ozlu et al. [43] showed no statistically significant differences between HILT and control interventions. Overall, these analyses indicated that the direction of the treatment effect for post-treatment pain outcomes remained consistent after exclusion of studies with higher RoB.

An additional sensitivity analysis excluding Eurcherdkul et al. [42], which used a superpulsed Nd:YAG laser system with parameters differing from the other included trials, was also conducted (Fig. 4E). After exclusion, the pooled analysis continued to show a statistically significant reduction in pain intensity in favor of HILT (SMD = − 0.72; 95% CI − 1.06 to − 0.38; EG = 68, CG = 83). Heterogeneity decreased to a moderate level (I^2^ = 48%), and the direction and magnitude of the effect remained consistent with the primary analysis, supporting the robustness of the findings.

#### Function

Three randomized controlled trials [40, 42, 43] (n = 103 participants) reporting functional outcomes using the Kujala Anterior Knee Pain Scale were included in the meta-analysis. In trials reporting multiple functional outcome measures (e.g., Kujala and LEFS in Ozlu et al. [42]), the Kujala scale was prioritized to ensure clinical and construct homogeneity across studies. Trials that did not report functional outcomes (e.g., Qayyum et al. [41]) were not included in the functional meta-analysis.

**Fig. 5:**
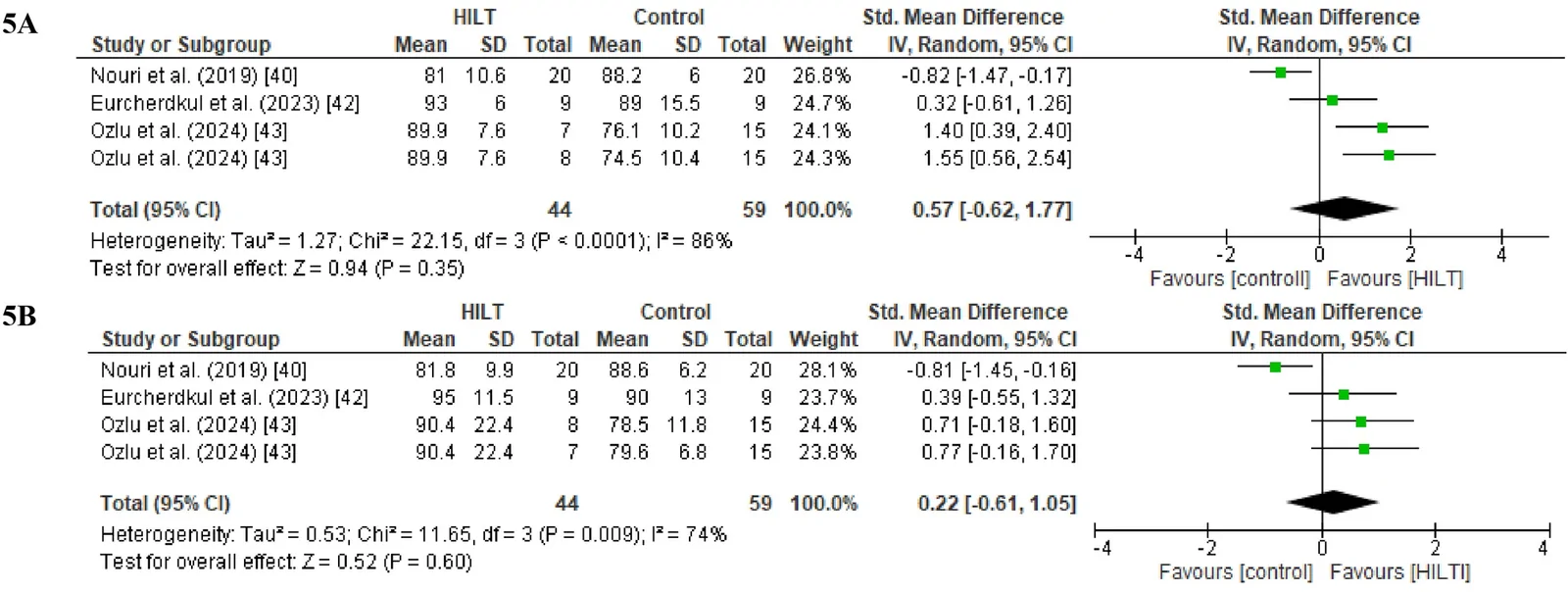
Forest plot for function at the end (**5A**) of treatment and at follow-up (**5B**) (Kujala scale): comparison of HILT with physical therapy interventions, including HILT placebo plus therapeutic exercise (strengthening and/or stretching), patellar mobilization, therapeutic ultrasound, transcutaneous electrical nerve stimulation (TENS), and interferential current therapy

At post-treatment (Fig. 5A), the pooled analysis showed a moderate but non-significant effect favoring HILT (SMD = 0.57; 95% CI − 0.62 to 1.77; EG = 44, CG = 59). At follow-up (4–12 weeks) (Fig. 5B), subgroup analysis demonstrated a small and non-significant effect (SMD = 0.22; 95% CI − 0.61 to 1.05; EG = 44, CG = 59).

According to the GRADE framework, functional outcomes were considered critical outcomes for patients [39]. The certainty of the evidence was rated as very low due to concerns related to risk of bias, inconsistency (I^2^ > 50%), and imprecision associated with the limited pooled sample size (Table 3) [39, 44]. Based on the predefined effect size thresholds, functional outcomes showed moderate clinical relevance at post-treatment and small clinical relevance at follow-up [37, 38].

#### Function sensitivity analysis

**Fig. 6:**
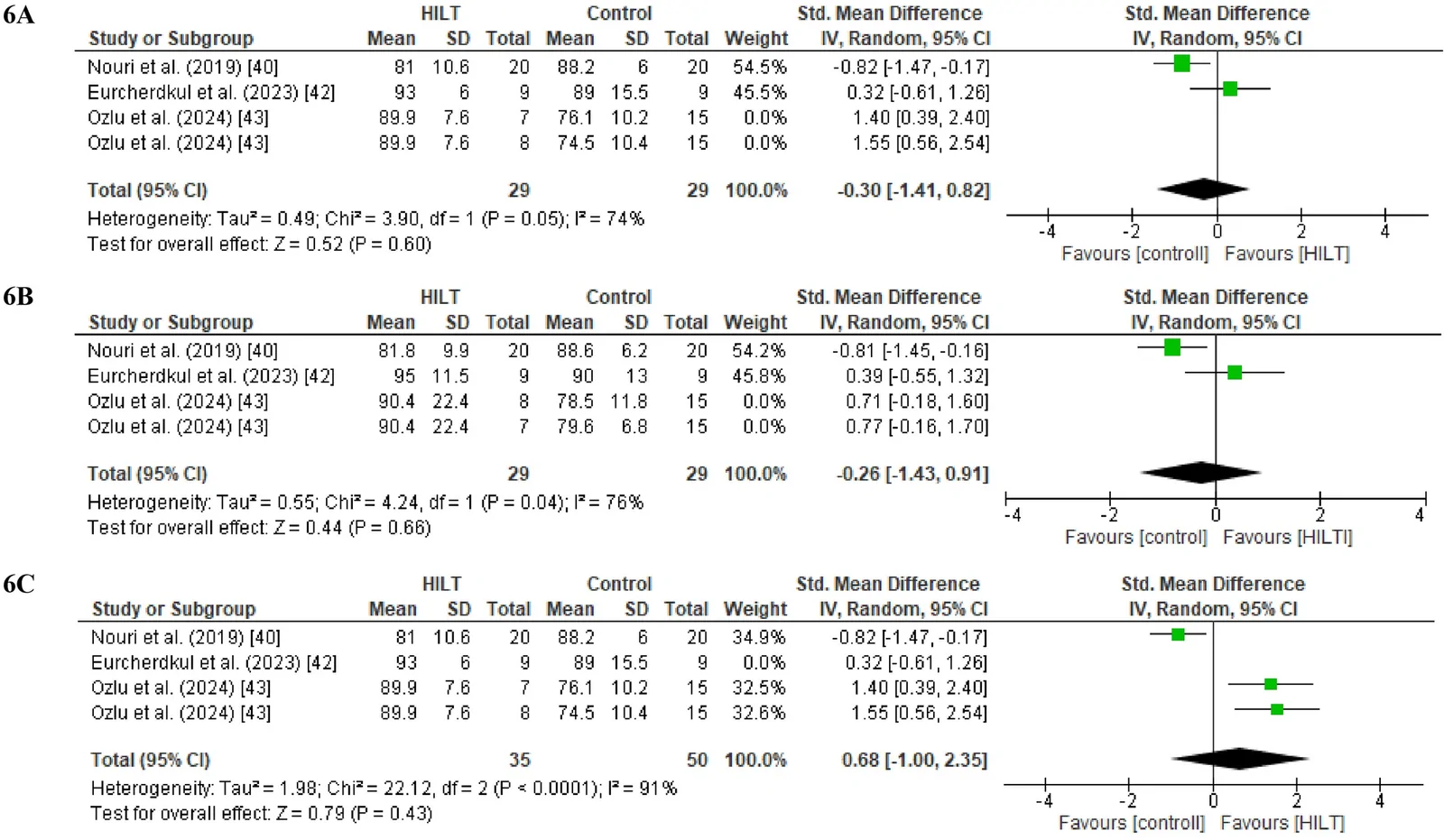
Forest plots of function assessed using the Kujala scale at the end of treatment (**6A**) and at follow-up (**6B**). Sensitivity analyses excluding Ozlu et al. [42] are shown for both post-treatment and follow-up assessments (**6A** and **6B**). An additional sensitivity analysis excluding Eurcherdkul et al. [42] (**6C**). The analyses compare HILT with other physical therapy interventions, including HILT placebo combined with therapeutic exercise (strengthening and/or stretching), patellar mobilization, therapeutic ultrasound, transcutaneous electrical nerve stimulation (TENS), and interferential current therapy

Sensitivity analyses were conducted by excluding the trial with the highest RoB according to the RoB 2 assessment (Ozlu et al. [43]) for functional outcomes at post-treatment (Fig. 6A) and at follow-up (4–12 weeks) (Fig. 6B). After exclusion of this study, no statistically significant differences were observed between HILT and control interventions at post-treatment (SMD = − 0.30; 95% CI − 1.41 to 0.82; EG = 29, CG = 29) or at follow-up (SMD = − 0.26; 95% CI − 1.43 to 0.91; EG = 29, CG = 29).

An additional sensitivity analysis excluding Eurcherdkul et al. [42], which used a superpulsed Nd:YAG laser system with parameters that differed from the other included trials, also showed no statistically significant difference between groups (SMD = 0.68; 95% CI − 1.00 to 2.35; EG = 35, CG = 50) (Fig. 6C). Heterogeneity remained high (I^2^ = 91%), indicating substantial variability across studies and supporting cautious interpretation of the pooled functional outcomes.

### Publication bias

Publication bias could not be statistically assessed due to the inclusion of fewer than 10 studies [45].

## Discussion

This systematic review evaluated the effects of HILT in patellofemoral pain (PFP), a condition for which evidence remains limited. Although studies in other musculoskeletal disorders describe HILT as a safe and non-invasive modality with analgesic and functional benefits [19–23], its role in PFP has not been clearly established. In this review, HILT combined with exercise-based rehabilitation was associated with reductions in pain intensity at post-treatment, with effects maintained at short- to medium-term follow-up (4–12 weeks). Although these findings suggest a potentially clinically meaningful analgesic effect, the very low certainty of the evidence and the absence of parallel improvements in function warrant cautious interpretation, as symptomatic pain relief may not necessarily translate into clinically meaningful functional gains. No adverse events were reported, in line with findings from other clinical populations [19–22, 46].

### HILT and pain relief

This review suggests that HILT, particularly when combined with therapeutic exercise, may reduce pain in people with PFP, with improvements not only evident at post-treatment but also maintained at short- to medium-term follow-up (4–12 weeks) [40–43]. Although the magnitude of the observed SMDs suggests a potentially meaningful reduction in pain at both time points, the very low certainty of the evidence—driven by methodological limitations, substantial heterogeneity, and imprecision reflected in wide confidence intervals, especially at follow-up—precludes confident conclusions regarding the true magnitude or durability of HILT’s analgesic effects. However, the clinical interpretability of these effects remains uncertain, as the observed changes cannot be directly translated into minimal clinically important differences due to the use of SMDs. Therefore, these findings should be interpreted as hypothesis-generating rather than practice-informing and should not be considered sufficient to support definitive clinical recommendations until RCTs with a low RoB become available.

The mechanisms underlying these effects are primarily related to photobiomodulation—activation of the electron transport chain, improved microcirculation, attenuation of oxidative stress, modulation of inflammatory pathways, reduced nociceptive conduction, and endogenous opioid release—together with photothermal actions [15, 17, 18]. Thermal effects may also contribute to analgesia by promoting muscle relaxation and interrupting the pain–spasm cycle, a self-perpetuating mechanism in which pain triggers reflex muscle contraction, further sustaining dysfunction. By disrupting this loop, therapy may help restore muscle homeostasis and facilitate functional recovery. Thermal effects have also been hypothesized to influence musculature affecting patellar alignment, potentially reducing excessive lateral tracking; however, this mechanism was not directly evaluated in the included trials [19, 47–49]. Unlike LLLT, HILT integrates both non-thermal and thermal mechanisms depending on mean power and is commonly applied using sequential scanning (thermal) and point (photobiomodulation) techniques. Its higher output power also allows delivery of greater energy in shorter treatment times and coverage of larger treatment areas [19, 20].

The effect sizes observed in this review (SMD − 0.66 post-treatment; SMD − 1.08 at follow-up) are broadly consistent with those reported for HILT in other musculoskeletal conditions. Although smaller than the pooled effects described for low back pain, neck pain, and other spinal disorders (SMDs 0.83–1.23) [19, 46, 50, 51], these comparisons should be interpreted cautiously given the limited number of available RCTs and methodological variability. In the only head-to-head comparisons available, HILT combined with exercise produced greater analgesic effects than ultrasound plus exercise and interferential current plus exercise, both used as active comparators. However, the low certainty of evidence means that conclusions regarding comparative superiority remain exploratory. Nevertheless, these findings are consistent with previous reports of HILT in knee conditions [52, 53] and may support its consideration as an adjunctive analgesic modality to enhance therapeutic exercise within multimodal rehabilitation strategies for PFP.

Pain reduction is particularly relevant in PFP, where quadriceps inhibition secondary to pain perpetuates patellar maltracking and joint overload. In addition, arthrogenic muscle inhibition caused by joint effusion or edema can further impair function [53]; the anti-inflammatory and anti-edematous effects of photobiomodulation may be especially valuable in these contexts [15, 17–19]. By restoring the conditions necessary for active rehabilitation, HILT acts as an effective adjunct to therapeutic exercise, which remains the cornerstone of PFP management. Strengthening of the vastus medialis, hip adductors, and abductors, combined with stretching of the hamstrings and iliotibial band—as consistently reported in RCTs—improves patellar alignment and reduces lateral joint stress [9, 55, 56]. This positioning is consistent with contemporary clinical practice guidelines for PFP, which emphasize exercise-based rehabilitation and patient education as the core components of management, while passive modalities are recommended only as adjuncts to facilitate pain reduction and participation in active rehabilitation [57]. Importantly, the present findings reinforce the principle that multimodal interventions, with exercise as the central component, achieve superior outcomes when complemented by analgesic modalities such as HILT [53]. Taken together, these findings support the interpretation that HILT primarily acts as an adjunctive analgesic modality, facilitating engagement in exercise-based rehabilitation rather than directly driving functional recovery.

The results also mirror evidence from LLLT combined with exercise, which has reported reductions in pain in PFP and knee osteoarthritis [16]. These converging findings suggest that photobiomodulation may represent the principal mechanism underlying the observed analgesic effects, particularly as most included studies applied point-based laser techniques [40–43]. However, the additional thermal component of HILT may partly explain its potential advantage over conventional electrotherapy modalities such as TENS or interferential currents, which act mainly through gate control and endorphin release. When combined with exercise, HILT may therefore provide broader therapeutic mechanisms, whereas the role of traditional electrotherapy in PFP remains uncertain and is not supported by current clinical practice guidelines [9].

Beyond peripheral and biomechanical mechanisms, emerging evidence highlights the relevance of psychosocial factors in pain modulation in PFP. In this context, an RCT by Bagheri et al. showed that adding mindfulness-based practice to exercise therapy resulted in greater pain reduction in female recreational runners with PFP, emphasizing the role of central and cognitive-emotional factors within rehabilitation strategies [58]. Similarly, a recent systematic review and meta-analysis by Naderi et al. reported that psychosocial interventions may reduce kinesiophobia in musculoskeletal rehabilitation, although the certainty of evidence remains limited [59].

### HILT and function

In contrast to its consistent analgesic effects, this review did not demonstrate statistically significant improvements in functional outcomes with HILT compared with other physical therapy interventions [19, 48–51]. Similar dissociations between symptom control and functional recovery have been reported in other patellofemoral conditions. For example, a recent systematic review comparing surgical and conservative management for first-time patellar dislocation showed that, despite improvements in the primary clinical outcome (redislocation rate), functional outcomes such as the Kujala score and quality-of-life measures were inconsistent and did not consistently favor the intervention associated with better structural or symptomatic control [60].

However, the absence of significant functional effects should be interpreted with caution. Functional outcomes were reported in only three randomized controlled trials, with fewer than 100 participants contributing to the pooled analyses (EG = 44; CG = 59), and substantial heterogeneity was observed (I^2^ > 50%). Under these conditions, the meta-analysis was likely underpowered to detect anything other than large effects, and therefore the current evidence is insufficient to conclude that HILT lacks functional benefits. Rather, the null findings may reflect limited statistical power and between-study variability, as outlined in methodological guidance for interpreting imprecision and small sample sizes in meta-analyses [35, 39].

This finding contrasts with evidence from other musculoskeletal conditions, such as knee osteoarthritis, where HILT has been associated with functional improvements. Nevertheless, given the small number of available RCTs in PFP and their methodological limitations, including heterogeneity in comparators and outcome measures, it remains premature to draw firm conclusions regarding the functional effects of HILT in this population.

The primary therapeutic action of HILT appears to be analgesia, with functional improvements likely arising indirectly through enhanced tolerance to exercise, which remains the cornerstone of PFP management. Functional restoration in PFP typically requires a broader multimodal strategy. Integrating HILT with exercise-based rehabilitation and, where appropriate, adjunctive interventions used in the included RCTs—such as patellar mobilization [9, 41, 55]—may help optimize outcomes within comprehensive rehabilitation programs. From a mechanistic perspective, evidence from other musculoskeletal pain conditions suggests that HILT may induce analgesic effects alongside modest improvements in objective functional parameters, reinforcing their role as adjuncts rather than stand-alone interventions [61].

This review evaluated function utilizing validated patient-reported outcome measures (PROMs), mainly the Kujala Anterior Knee Pain Scale and, in one instance, the WOMAC Index. While these PROMs provide clinically meaningful assessments of patient-perceived disability, future trials should consistently incorporate standardized PROMs alongside objective performance-based measures. Importantly, adequately powered RCTs with sufficient sample sizes and standardized functional outcomes are needed to determine whether HILT confers functional benefits when used as an adjunct to exercise-based rehabilitation in PFP.

### HILT parameters for treating PFP

Based on the dosimetric parameters reported in the included RCTs [40–43], a pragmatic HILT posology for PFP can be proposed. A wavelength of 1064 nm was consistently used, with mean power levels of approximately 8–10 W and energy densities around 100–120 J/cm^2^, delivering total energies of 1000–1500 J over 2–5 min of treatment. Continuous emission was most frequently applied, although pulsed or combined modes may offer additional flexibility depending on therapeutic goals, enhancing photobiomodulation alone or together with thermal effects. Point application was typically used to deliver targeted photobiomodulation, whereas larger applicators were applied in scanning mode to induce thermal effects.

In parallel, therapeutic exercise and patient education remain the cornerstone of PFP rehabilitation [8, 12]. RCTs consistently emphasize strengthening of the vastus medialis and hip adductors and abductors, combined with stretching of the hamstrings and iliotibial band, to improve patellar alignment and reduce lateral overload [12, 40–43, 55]. These interventions address key biomechanical contributors to PFP and contribute to improved long-term outcomes [55, 56]. Current best practice guidelines also highlight patient education as a fundamental component of management, helping patients understand their condition, set realistic expectations, support load management, and reduce fear associated with movement [12, 55, 56].

### Clinical perspective

From a rehabilitation perspective, multimodal strategies are commonly adopted to address the complex and multifactorial nature of knee pain, particularly in physically active populations [5–7, 56, 59, 62, 63]. Previous evidence indicates that integrated rehabilitation approaches combining exercise with adjunctive interventions can achieve clinically relevant short-term pain reduction in people with anterior knee pain [56]. Consistent with this framework, all RCTs included in this review combined HILT with strengthening and/or stretching exercises [40–43], reflecting the complementary roles of analgesic modalities in pain modulation and therapeutic exercise in addressing biomechanical and neuromuscular contributors to symptoms [8, 59, 62, 64]. Within this context, the analgesic effects observed in this review should be interpreted as the specific contribution of HILT within exercise-based rehabilitation, rather than as evidence supporting HILT as an isolated therapeutic approach. Such multimodal conservative approaches are routinely applied in musculoskeletal practice by various health professionals, including physical therapists and chiropractors, where physical agents such as HILT may be used as adjuncts to manual therapy, exercise, and patient education [9–11].

### Study limitations

To our knowledge, this is the first systematic review specifically addressing HILT in PFP, highlighting both the novelty of the topic and the limited available evidence. This review has several limitations that should be considered when interpreting the findings.

The most significant limitation of this review is the inability to isolate the independent therapeutic contribution of HILT, as all included RCTs delivered HILT exclusively in combination with exercise-based rehabilitation. Consequently, the observed pooled effects reflect the combined intervention rather than HILT alone, which limits causal attribution and clinical inferences regarding the standalone value of HILT.

The number of available RCTs was small, which reduced the precision of pooled estimates and limited the ability to conduct subgroup or sensitivity analyses. This limited precision was further reflected in wide confidence intervals around pooled effect sizes, indicating substantial uncertainty regarding the true magnitude of treatment effects. Considerable heterogeneity in comparator interventions (e.g., ultrasound plus exercise, TENS, interferential current, patellar mobilization) further constrained the interpretability of pooled effects, particularly for functional outcomes. Moreover, although pain intensity was the primary outcome showing statistically significant effects, improvements in pain were not consistently accompanied by parallel functional gains, which further limits the clinical interpretability of the observed analgesic effects. Variability in HILT protocols—particularly in delivered energy, reporting of mean output power versus nominal peak power, and application techniques—limited direct comparison of HILT dosing across studies and precluded a detailed dose–response analysis. Nevertheless, the present synthesis provides a pragmatic overview that may help inform the design and reporting of future clinical trials.

Methodological shortcomings were evident, including inadequate blinding and selective reporting, which may have introduced bias and contributed to the overall low certainty of evidence. Small sample sizes and short follow-up periods in some RCTs further limited the strength of inferences regarding both the magnitude and durability of treatment effects. Although symptom chronicity was controlled a priori through the eligibility criteria (minimum duration ≥ 3 months), the exact duration of symptoms beyond this threshold and some baseline clinical characteristics were not consistently reported across trials, limiting a more granular assessment of population homogeneity. Reporting of adverse events and detailed HILT parameters was inconsistent, reducing reproducibility and hindering assessment of treatment safety.

Together, these limitations highlight the need for larger, methodologically rigorous RCTs with standardized HILT protocols, consistent use of validated outcomes, and longer follow-up to better determine the role of HILT in PFP management.

### Future directions

Future research on HILT for PFP should address several key shortcomings, particularly small sample sizes and insufficient blinding procedures. Future trials should be designed to disentangle the effects of HILT from those of exercise-based rehabilitation. In practice, this will likely require multi-arm RCTs that include an exercise-only group, a HILT-only group, and a combined HILT-plus-exercise group, allowing estimation of HILT’s independent effect and any interaction with exercise. This approach is consistent with methodological guidance for the evaluation of complex interventions, which emphasizes the importance of identifying active components and their interactions when interventions are delivered in combination [60]. To improve comparability across studies and strengthen clinical applicability, future trials should consistently use validated outcome measures, such as the VAS for pain and the Kujala or WOMAC scales for function, as heterogeneous instruments limit interpretability. Greater methodological rigor and standardized dosimetric protocols—such as the pragmatic framework synthesized in this review—will be essential to generate higher-quality evidence and determine whether HILT provides meaningful added value in PFP rehabilitation.

## Conclusion

This systematic review is, to our knowledge, the first to focus specifically on the effects of HILT in people with PFP. The available evidence indicates that HILT, when delivered alongside exercise-based rehabilitation, is associated with reductions in pain intensity compared with other physical therapy interventions, including electrophysical agents combined with exercise, exercise alone, or exercise plus placebo HILT. However, the very low certainty of the evidence precludes confident conclusions regarding the true magnitude or durability of this effect, and its impact on functional outcomes remains uncertain.

The certainty of these findings is limited by small sample sizes, methodological weaknesses, high RoB, and heterogeneity in comparator interventions, highlighting important gaps in the current literature. By synthesizing the available dosimetric parameters, this review provides a pragmatic framework that may inform the design and reporting of future clinical trials. Given the very low certainty of evidence, no definitive clinical recommendations can currently be made regarding the use of HILT—either as a standalone or adjunctive intervention—for the management of PFP. The observed pain reductions should therefore be interpreted as hypothesis-generating, requiring confirmation through adequately powered, methodologically rigorous RCTs employing validated outcome measures before any clinical implementation can be considered. Until such evidence is available, exercise-based rehabilitation remains the only intervention supported by higher-certainty evidence for PFP management.

Future research should focus on RCTs specifically designed to address the methodological limitations identified in this review. Such studies should incorporate improved blinding procedures, standardized and transparently reported HILT dosimetric protocols, and longer follow-up periods to evaluate the durability of treatment effects. These design features are necessary to clarify the clinical relevance of HILT and to determine whether pain reductions are accompanied by sustained functional improvements in patients with PFP.

## Supplementary Information

Below is the link to the electronic supplementary material.

Supplementary Table 1. Search strategy (last updated May 1, 2026)

Supplementary Table 2. Summary of excluded articles

Supplementary Table 3. Methodological quality of the included studies (PEDro scale)

## Data Availability

The datasets used and/or analysed during the current study are available from the corresponding author on reasonable request.
