# Supplementary material for: Efficacy of high-intensity laser therapy for patellofemoral pain: a systematic review and meta-analysis
Source: Chiropr Man Therap. 2026 Jun 12;34:33. doi: 10.1186/s12998-026-00653-z (PMC13487960; doi:10.1186/s12998-026-00653-z)
Supplement: Supplementary file 1 [file 12998_2026_653_MOESM1_ESM.docx]

**Supplementary Table 1.** Search strategy (last updated May 1, 2026).

| Search strategy | | Identification of studies via databases and registers | | | | | | | | | Identification of studies via other methods |
| --- | --- | --- | --- | --- | --- | --- | --- | --- | --- | --- | --- |
| Keywords | | PubMed | Scopus | Web of Science | CINAHL | Medline | ScienceDirect | Cochrane Library | PEDro | **TOTAL** | Google Scholar |
| S1 | *"Lasers"* | 103,462 | 1,794,790 | 1,311,517 | 11,178 | 103,113 | 1,000,000 | 28,082 |  | 4,352,142 |  |
| S2 | *"Laser Therapy"* | 52,859 | 52,045 | 16,502 | 13,146 | 53,728 | 24,649 | 8,365 |  | 221,294 |  |
| S3 | *"High-Intensity Laser Therapy"* | 224 | 317 | 342 | 120 | 224 | 135 | 275 |  | 1,637 |  |
| S4 | *"High-power laser"* | 1,440 | 22,526 | 9,196 | 58 | 1,438 | 21,006 | 139 |  | 55,803 |  |
| S5 | *"Class IV laser"* | 36 | 67 | 147 | 21 | 36 | 174 | 39 |  | 520 |  |
| S6 | S1 OR S2 OR S3 OR S4 OR S5 | 140,749 | 1,794,790 | 199,690 | 21,636 | 140,947 | 1,045,964 | 28,074 |  | 3,371,850 |  |
| S7 | *"Musculoskeletal Pain"* | 13,691 | 23,281 | 16,164 | 5,871 | 13,638 | 26,520 | 3,579 |  | 102,744 |  |
| S8 | *"Musculoskeletal diseases"* | 20,809 | 55,734 | 4,092 | 13,838 | 25,032 | 15,303 | 6,110 |  | 140,918 |  |
| S9 | *"Knee Joint"* | 87,511 | 99,168 | 31,749 | 31,288 | 92,867 | 84,134 | 10,296 |  | 437,013 |  |
| S10 | *"Patellofemoral Pain Syndrome"* | 1,890 | 2,812 | 1,423 | 2,046 | 1,899 | 1,703 | 900 |  | 12,673 |  |
| S11 | *"Patellofemoral Joint"* | 4,414 | 8,159 | 3,187 | 19,278 | 49,471 | 6,779 | 422 |  | 91,710 |  |
| S12 | *"Pain Management"* | 97,133 | 78,432 | 48,733 | 45,759 | 108,751 | 99,809 | 20,031 |  | 498,648 |  |
| S13 | S7 OR S8 OR S9 OR S10 OR S11 OR S12 | 218,153 | 255,799 | 102,445 | 95,455 | 238,941 | 217,602 | 39,536 |  | 1,167,931 |  |
| S14 | S7 OR S14 | **830*** | **2,025*** | **442*** | **456*** | **902*** | **641*** | **607*** | **3*** | **5,903** | **1960**** |

*Search algorithm used for formal databases: *("Lasers" OR "Laser Therapy" OR "High-Intensity Laser Therapy" OR "High-power laser" OR "Class IV laser") AND ("Musculoskeletal Pain" OR "Musculoskeletal diseases" OR "Knee Joint" OR "Patellofemoral Pain Syndrome" OR "Patellofemoral Joint" OR "Pain Management")* **Search algorithm for other identification of studies via other methods: *("High-Intensity Laser Therapy" OR "High-power laser" OR "Class IV laser") AND ("Musculoskeletal Pain" OR "Patellofemoral Pain Syndrome" OR "Patellofemoral Joint" OR "Pain Management")*
