# Supplementary material for: Efficacy of high-intensity laser therapy for patellofemoral pain: a systematic review and meta-analysis
Source: Chiropr Man Therap. 2026 Jun 12;34:33. doi: 10.1186/s12998-026-00653-z (PMC13487960; doi:10.1186/s12998-026-00653-z)
Supplement: Supplementary file 2 [file 12998_2026_653_MOESM2_ESM.docx]

**Supplementary Table 2.** Summary of excluded articles via databases, registries, and other methods.

| N° | Source | Author (year) | Reference | Reason of exclusion |
| --- | --- | --- | --- | --- |
| 1 | Studies via database and registers | Allam et al. (2025) | Allam NM, Alsirhani H, Alruwaili MB, Dosh DM, Alruwaili HM, Almazyad WH, et al. Effect of laser acupuncture on pain, range of motion, and function in patellofemoral pain syndrome: a randomised controlled trial. Front Med (Lausanne). 2025;12(1613197):1613197. http://dx.doi.org/10.3389/fmed.2025.1613197 | LLLT in PFPS |
| 2 | Studies via database and registers | Pocai et al. (2021) | Pocai, B. L., Provensi, É., Serighelli, F., Rigo, G., Artioli, D. P., de Albuquerque, C. E., & Bertolini, G. R. F. (2021). Effect of photobiomodulation in the patellofemoral pain syndrome; randomized clinical trial in young women. Journal of Bodywork and Movement Therapies, 26, 263–267. https://doi.org/10.1016/j.jbmt.2021.01.003 | LLLT in PFPS |
| 3 | Studies via database and registers | ElShamy et al. (2018) | El-Shamy SM, Abdelaal AAM. Efficacy of pulsed high-intensity laser therapy on pain, functional capacity, and gait in children with haemophilic arthropathy. Disabil Rehabil. 2018;40(4):462–8. http://dx.doi.org/10.1080/09638288.2016.1261416 | Haemophilic arthropathy |
| 4 | Studies via database and registers | Sedighi-Pashaki et al. (2018) | Sedighi-Pashaki, Mohammad, et al. "Design of a Clinical Trial to Determine the Effects of Low Level Laser on Pain and Function in Patellofemoral Pain Syndrome: Protocol for a Triple-Blinded Randomized Clinical Trial." Journal of Research in Rehabilitation Sciences 14.5 (2018): 303-310. | LLLT in PFPS |
| 5 | Studies via database and registers | Šifta et al. (2015) | Šifta P, Danilov D. Effects of high-intensity laser on gonarthrosis. 2015 [cited 2025 Sept 4]; Available from: https://www.asalaser.com/sites/default/files/ricerca/documenti/e4h14_effect_high_intensity_laser_gonarthrosis.pdf | Gonarthrosis |
| 6 | Studies via database and registers | Rogvi-Hansen et al. (1991) | Rogvi-Hansen B, Ellitsgaard N, Funch M, Dall-Jensen M, Prieske J. Low level laser treatment of chondromalacia patellae. Int Orthop. 1991;15(4):359–61. http://dx.doi.org/10.1007/bf00186879 | LLLT in chondromalacia patellae |

**Abbreviations:** LLLT, Low-Level Laser Therapy; HILT, High-Intensity Laser Therapy; PBM, Photobiomodulation; PFPS, Patellofemoral Pain Syndrome; ROM, Range of Motion; RCT, Randomized Controlled Trial.
