# Supplementary material for: Efficacy of high-intensity laser therapy for patellofemoral pain: a systematic review and meta-analysis
Source: Chiropr Man Therap. 2026 Jun 12;34:33. doi: 10.1186/s12998-026-00653-z (PMC13487960; doi:10.1186/s12998-026-00653-z)
Supplement: Supplementary file 3 [file 12998_2026_653_MOESM3_ESM.docx]

**Supplementary Table 3.** Methodological quality of the included studies assessed with the PEDro scale.

| **Author (year) Country** | **PEDro scale criteria** | | | | | | | | | | | **Total score** |
| --- | --- | --- | --- | --- | --- | --- | --- | --- | --- | --- | --- | --- |
|  | **Criteria 1*** | **Criteria 2** | **Criteria 3** | **Criteria 4** | **Criteria 5** | **Criteria 6** | **Criteria 7** | **Criteria 8** | **Criteria 9** | **Criteria 10** | **Criteria 11** |  |
| Nouri et al. (2019)** [38] Iran | Y | Y | N | Y | Y | N | N | Y | N | Y | Y | **6/10** |
| Qayyum et al. (2022)** [39] Pakistan | Y | Y | N | Y | N | N | N | N | N | Y | Y | **4/10** |
| Eurcherdkul et al. (2023)^+^ [40] Thailand | Y | Y | N | Y | Y | N | Y | Y | Y | Y | Y | **8/10** |
| Ozlu et al. (2024)** [41] Turkey | N | Y | N | Y | N | N | N | Y | N | Y | Y | **5/10** |

**PEDro (Physiotherapy Evidence Database) scale criteria:**

(1) Eligibility criteria were specified; (2) Random allocation; (3) Concealed allocation; (4) Baseline comparability; (5) Blind subjects; (6) Blind therapists; (7) Blind assessors; (8) Adequate follow-up; (9) Intention-to-treat analysis; (10) Between-group comparisons; (11) Point estimates and variability

*Eligibility criteria item does not contribute to the total score

**Score confirmed by the PEDro database

+ Score determined by researchers (Not available in PEDro database)
